# Factors that Influence the Reported Sensitivity of Rapid Antigen Testing for SARS-CoV-2

**DOI:** 10.3389/fmicb.2021.714242

**Published:** 2021-10-05

**Authors:** Valentin Parvu, Devin S. Gary, Joseph Mann, Yu-Chih Lin, Dorsey Mills, Lauren Cooper, Jeffrey C. Andrews, Yukari C. Manabe, Andrew Pekosz, Charles K. Cooper

**Affiliations:** ^1^Becton, Dickinson and Company, BD Life Sciences–Integrated Diagnostic Solutions, Sparks, MD, United States; ^2^W. Harry Feinstone Department of Molecular Microbiology and Immunology, Johns Hopkins Bloomberg School of Public Health, Baltimore, MD, United States; ^3^Department of Medicine, Johns Hopkins University School of Medicine, Baltimore, MD, United States; ^4^Department of Emergency Medicine, Johns Hopkins University School of Medicine, Baltimore, MD, United States

**Keywords:** test sensitivity, SARS-CoV-2, diagnostic accuracy, rapid antigen testing, RT-PCR, meta-regression analysis, systematic review and meta-analysis, viral culture

## Abstract

Tests that detect the presence of severe acute respiratory syndrome coronavirus-2 (SARS-CoV-2) antigen in clinical specimens from the upper respiratory tract can provide a rapid means of coronavirus disease 2019 (COVID-19) diagnosis and help identify individuals who may be infectious and should isolate to prevent SARS-CoV-2 transmission. This systematic review assesses the diagnostic accuracy of SARS-CoV-2 antigen detection in COVID-19 symptomatic and asymptomatic individuals compared to quantitative reverse transcription polymerase chain reaction (RT-qPCR) and summarizes antigen test sensitivity using meta-regression. In total, 83 studies were included that compared SARS-CoV-2 rapid antigen-based lateral flow testing (RALFT) to RT-qPCR for SARS-CoV-2. Generally, the quality of the evaluated studies was inconsistent; nevertheless, the overall sensitivity for RALFT was determined to be 75.0% (95% confidence interval: 71.0–78.0). Additionally, RALFT sensitivity was found to be higher for symptomatic vs. asymptomatic individuals and was higher for a symptomatic population within 7 days from symptom onset compared to a population with extended days of symptoms. Viral load was found to be the most important factor for determining SARS-CoV-2 antigen test sensitivity. Other design factors, such as specimen storage and anatomical collection type, also affect the performance of RALFT. RALFT and RT-qPCR testing both achieve high sensitivity when compared to SARS-CoV-2 viral culture.

## Introduction

Severe acute respiratory syndrome coronavirus-2 (SARS-CoV-2) is the highly transmissible viral agent responsible for the development of coronavirus disease 2019 (COVID-19; Carlos et al., 2020; Chang et al., 2020; Li et al., 2020; Wang et al., 2020). Based on measurements from specimen swabs, the viral load in infected individuals peaks around the time of symptom onset (approximately 2–3 days following infection; Walsh et al., 2020). This time point coincides with the highest rate of SARS-CoV-2 transmissibility. Transmissibility usually tapers off within 8 days following symptom onset (He et al., 2020). Asymptomatic individuals account for 40–45% of all infections and can transmit the virus for up to 14 days following infection (Oran and Topol, 2020). Therefore, rapid accurate diagnostic testing has been a key component of the response to COVID-19, as identification of SARS-CoV-2-positive individuals facilitates both appropriate treatment and reduced communal spread of the virus (La Marca et al., 2020).

Molecular testing using quantitative reverse transcription polymerase chain reaction (RT-qPCR) platforms has become the primary diagnostic method for COVID-19 diagnosis (Wang and Taubenberger, 2010; Food and Drug Administration, 2011; Centers for Disease Control, 2020; Tahamtan and Ardebili, 2020). The major advantage of RT-qPCR testing is its high analytical sensitivity (translating to few false-negative results; Giri et al., 2021). However, large-scale clinical laboratory testing requires a dedicated infrastructure and specialized technician training. In addition, due to the specimen transport and processing time, results for standard RT-qPCR can take days to obtain, depending on the catchment area and the demand for testing (Bustin and Nolan, 2020).

**FIGURE 1:**
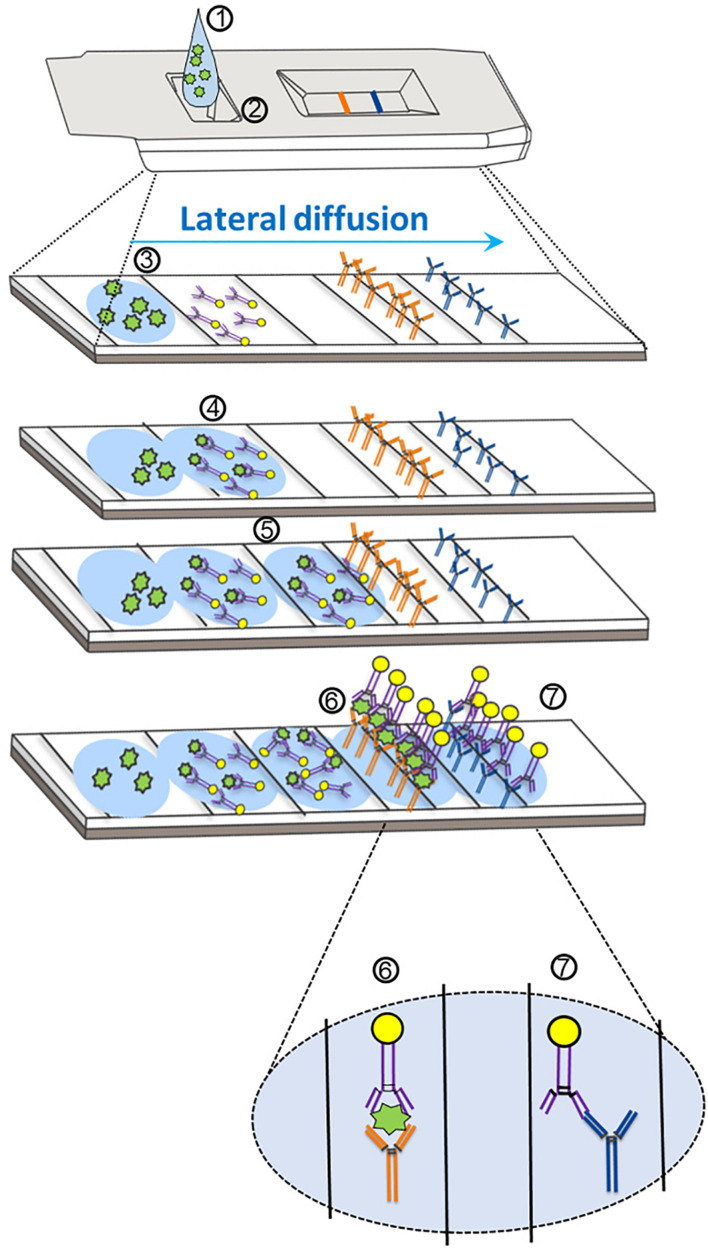
Mechanism of action for severe acute respiratory syndrome coronavirus-2 (SARS-CoV-2) antigen detection through lateral flow assay design. (1) The specimen analyte, containing SARS-CoV-2 antigen suspended in assay buffer, is deposited in the sample well (at 2). (3) The analyte (containing antigen; in green) is absorbed into the sample pad and begins to diffuse across the reaction chamber into the conjugate pad. (4) The analyte comes into proximity of a SARS-CoV-2-specific antigen antibody that is conjugated to a tag (usually consisting of gold, latex, or a fluorophore). (5) The antigen–antibody complex migrates via diffusion across the nitrocellulose membrane. (6) The SARS-CoV-2 antigen–antibody complex comes into proximity of a second SARS-CoV-2 antigen antibody (different epitope) that is covalently bound to the device pad, and an antibody–antigen–antibody complex forms, resulting in the test line. Further diffusion of excess SARS-CoV-2 antibodies (unbound to antigen) results in association of a second covalently bound antibody that is specific for the first SARS-CoV-2 antibody. (7) An antibody–antibody complex forms resulting in the control line.

Antigen-based testing involves the application of specific SARS-CoV-2 antibodies (Figure 1) in several formats, including lateral flow immunofluorescent sandwich assays, chromatogenic digital immunoassay, lateral flow immunoassay with visual read, and microfluidic immunofluorescence assays (Rezaei et al., 2020). Antigen testing for SARS-CoV-2 can be utilized either in conjunction with RT-qPCR as a first-line screening test or in decentralized health care settings in which RT-qPCR testing may not be conducive for rapid result turnaround (Rezaei et al., 2020). Rapid antigen-based lateral flow testing for SARS-CoV-2, as with influenza, has been implemented globally to achieve rapid accurate results for COVID-19 diagnosis (Peeling et al., 2021). Although the majority of antigen-based tests share a common mechanism for detection of SARS-CoV-2 protein, the reported sensitivities of both Food and Drug Administration (FDA) Emergency Use Authorization (EUA)-approved and non-EUA-approved antigen-based tests have varied greatly in the literature (Brümmer et al., 2021). Multiple meta-analyses and systematic reviews have reported large inter-study heterogeneity related to SARS-CoV-2 antigen-based testing (Dinnes et al., 2020; COVID-19 Scientific Advisory Group Rapid Evidence Report, 2020; Brümmer et al., 2021). Although reliable antigen test performance coincides with a high specimen viral load (Brümmer et al., 2021), study heterogeneity could impact our conclusions about antigen test performance. Factors that could affect overall antigen test performance include *analytical sensitivity* (i.e., antibody/antigen binding affinity) of the assay, which likely varies for tests across manufacturers (Mina et al., 2020), *biases* occurring from the study design (e.g., blinding, test order, etc.), the *study population* [e.g., symptomatic vs. asymptomatic, days from symptom onset (DSO), etc.], the *anatomical collection site* (e.g., nasopharyngeal vs. anterior nares), and *specimen storage conditions* (Lijmer et al., 1999; Griffith et al., 2020; Accorsi et al., 2021).

As others have noted previously, a wide range of reported sensitivities has been documented for rapid antigen testing (Dinnes et al., 2020; Brümmer et al., 2021). The main objective of this meta-analysis was to explore possible causes of the high degree of heterogeneity of assay sensitivity estimates across different studies. Data were summarized and analyzed from over 80 articles and manufacturer instructions for use (IFU) to provide results on sensitivity for SARS-CoV-2 antigen testing from more than 25 individual assays.

## Materials and Methods

The methods for conducting research and reporting results for systematic reviews and meta-analyses, which are outlined by the Cochrane Collaboration Diagnostic Test Accuracy Working Group and by Preferred Reporting Items for Systematic reviews and Meta-Analyses (PRISMA) guidelines, were employed for this study (Gatsonis and Paliwal, 2006; Leeflang, 2014; Page et al., 2020). This study protocol was registered with the PROSPERO International Prospective Register of Systematic Reviews in 2021 (PROSPERO CRD42021240421; Booth et al., 2011).

The PICO (Participants, Intervention, Comparator, and Outcomes) of this meta-analysis was as follows: Participants were individuals undergoing SARS-CoV-2 testing in a healthcare setting (at least eight cases); Intervention (primary) was the index test consisting of a SARS-CoV-2 antigen detection platform utilizing immunobiological mechanisms, such as a sandwich ELISA, combined with spatial resolution (e.g., immunochromatographic assay); Intervention (secondary) was testing for SARS-CoV-2 using antigen and RT-qPCR testing (indices 1 and 2); Comparator (primary) was RT-qPCR as the reference test for detecting SARS-CoV-2 genomic RNA (any target gene); Comparator (secondary) was SARS-CoV-2 viral culture as the reference method for identifying specimens with infectious viral particles; and Outcome was the determination of antigen test sensitivity across independent variables.

### Search and Selection Criteria

Eligible studies/sources included diagnostic studies of any design type (i.e., retrospective, prospective, randomized, blinded, and non-blinded) that specifically involved the detection of SARS-CoV-2. The primary outcome was sensitivity for the detection of SARS-CoV-2 in a healthcare setting by rapid antigen testing as compared with RT-qPCR. Both MEDLINE and MedRxiv electronic databases were searched across dates ranging from January 1, 2020, to February 1, 2021, with the following search terms: (1) [(Antigen test and (sars-cov-2 OR COVID-19)] OR ((antigen[title/abstract] AND test) OR (Antigen[title/abstract] and assay)) AND (SARS-CoV-2[title/abstract] OR COVID-19[title/abstract])) and (2) ‘‘SARS-CoV-2 and antigen test or COVID-19 and antigen test.’’ In addition, a search was performed on the FDA database^1^ for all EUA SARS-CoV-2 antigen tests. All retrieved sources were assessed for relevance using predetermined inclusion/exclusion criteria. The inclusion criteria consisted of the following: (1) SARS-CoV-2 diagnostic target; (2) Sensitivity as a performance outcome; (3) Compares antigen testing performance with RT-qPCR as reference; (4) Population includes symptomatic and/or asymptomatic participants; (5) Human study; (6) English language; and (7) Any region, country, or state. Secondary inclusion subcriteria for analyses included the following: (S1) Index performance results that were stratified by viral load or by RT-qPCR (reference) cycle threshold (Ct); (S2) Delineated specimens for reference and index testing between symptomatic and asymptomatic participants; (S3) Delineated the anatomical site for specimen collection prior to reference and index testing; (S4) Delineated whether the specimen was frozen prior to reference testing; (S5) Specified whether the specimen was frozen prior to index testing; (S6) Analytical limit of detection (LOD) information was available for the reference assay; and (S7) The index test manufacturer information was available. The exclusion criteria included the following: (1) Article/source from a non-credible source; (2) Article/source contains an unclear or indistinct research question; (3) Does not contain performance data specific to SARS-CoV-2; (4) Does not identify or does not involve standard upper respiratory SARS-CoV-2 specimens (e.g., contains other specimen types such as serum or saliva); (5) Contains no RT-qPCR reference results for comparison; (6) Data were collected in an unethical manner; (7) The index test involves a mechanism other than SARS-CoV-2 antigen detection involving a lateral flow (or similar) design; (8) Data not conducive for extraction required for analysis; and (9) No data regarding true-positive and false-negative rates for the index test relative to the reference test. Additional secondary exclusion criteria included (S1) Article/source not in the English language; and (S2) Study did not involve humans.

Full-text reviews of the articles that passed initial screening were performed to identify sources that met inclusion/exclusion criteria involving study methodologies, specimen collection, SARS-CoV-2 test details, data type (sensitivity, specificity values, etc.) and format [raw data, only point estimates and 95% confidence intervals (95% CI) included, etc.]. The information was then entered into data extraction tables to document study characteristics and to record raw data with calculated point estimates and 95% CIs. A modified Newcastle–Ottawa Scale was used to evaluate the risk of bias (individual study quality; Wells et al., 2011), which included the following bias domains: detection (measurement of test result), reporting (failure to adequately control confounding, failure to measure all known prognostic factors), and spectrum (eligibility criteria, forming the cohort, and selection of participants). Risk of bias summary assessments for individual studies were categorized as “high,” “moderate,” or “low.” The overall quality of evidence for the risk estimate outcomes (all included studies) was obtained using a modified Grading of Recommendations, Assessment, Development and Evaluation (Schunemann et al., 2013) methodology for observational diagnostic studies.

The seven domains used to ascertain the overall study quality and strength across the six independent variables were (1) Confounder effect; (2) Consistency; (3) Directness; (4) Magnitude of effect; (5) Precision; (6) Publication bias; and (7) Risk of bias (ascertained from individual studies). Study subgroups were considered high quality when ≥4 of seven domains received a green rating, with no red ratings, and <3 unclear ratings; otherwise, it was considered moderate quality. Study subgroups were considered moderate quality when three domains were green with <3 red domains; or when two domains were green and <3 domains were red with <4 domains unclear; or when one domain was green with <2 red domains and <3 domains were unclear; or when no domains were green, no domains were red, and <2 domains were unclear. Any other combination of ratings resulted in a classification of quality as low.

Subgroup meta-analysis was performed for the following factors: (1) viral load with fixed cutoff values; (2) symptomatic vs. asymptomatic; (3) ≤7 DSO vs. any DSO; (4) anatomical collection type for specimens used for both index and reference testing (anterior nares/mid-turbinate vs. nasopharyngeal/oropharyngeal); (5) specimen storage conditions (fresh vs. frozen); (6) analytical sensitivity of the reference RT-qPCR test [detection cutoff < 500 genomic copies/ml (cpm) vs. ≥500 cpm]; and (7) assay manufacturer.

### Data Analysis

Data extraction was accomplished by two reviewers/authors with any discrepancies adjudicated by a third reviewer/author. An independent author performed all statistical methods. All analyses were performed using R software (version 4.0.2) (R Core Team, 2020) along with the meta (Balduzzi et al., 2019) and metaphor (Viechtbauer, 2010) packages. For each study, the sensitivity of the index test along with 95% Clopper–Pearson CI was calculated. Logit-transformed sensitivity values were combined to obtain random effect estimates of overall sensitivity. The same method was applied to subgroup meta-analyses; subgroups were defined by disease status, reference and test collection type, reference and test storage (fresh/frozen), study spectrum bias, reference analytical sensitivity (high and low), and manufacturer. *Q-tests* for heterogeneity based on random-effects models with common within-group variability were used to evaluate statistical differences between subgroups (univariate analysis). Moderators with significant heterogeneity in the subgroup analysis were included in a meta-regression mixed-effects model. Forest plots were generated for all subgroup analyses; a funnel plot of all logit-transformed sensitivities was generated without taking into account study characteristics, and another funnel plot of residual values was generated after fitting the meta-regression model. Separately, for articles where viral load information was available, subgroup meta-analysis by viral load (either measured by RT-PCR Ct of 25 or 30 or a viral cpm of 1 × 10^5^) and symptomatic status was performed. The minimum number of studies required for synthesis is *n* = 3.

## Results

**FIGURE 2:**
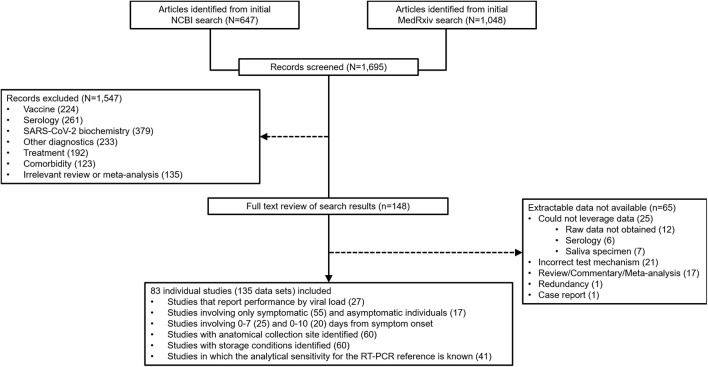
Preferred Reporting Items for Systematic reviews and Meta-Analyses (PRISMA) flow diagram for reconciliation of articles/sources included in this study.

At the outset, 1,695 sources were identified during the database search (Figure 2). From that group of candidate sources, screening was performed by title and abstract, and the potential pool of articles was reduced, and 148 underwent full-text review for data extraction. 83 articles/sources of the 148 were chosen for meta-analysis (Table 1) based upon further exclusion criteria (see section “*Materials and Methods”*). Note that a list of excluded sources with their specific exclusion criteria is available upon request from the authors. Of the 83 source studies, 76 (91.6%) involved a cross-sectional study design. Data from 12 of the sources were from validation studies as part of EUA from the FDA and can be found in each, respective, manufacturer IFU. 22 of the studies were conducted in the United States, nine in Spain, seven each from Germany and Japan, six from Italy, four from China, three each from France, Switzerland, and the United Kingdom, and two each from Belgium and Chile; the rest of the countries represented in this study had only one. 135 individual data sets were utilized in total from the 83 articles/sources; 30 articles provided more than one data set. The overall combined number of specimens from participants, across all 83 studies, was 53,689; the overall total number of RT-qPCR reference positive results for estimating sensitivity from all 135 data sets was 13,260.

**TABLE 1:** Information characterizing data sources involving SARS-CoV-2 antigen testing for analyses described in this report.

RFID	Source RFID	Country	Total N	Reference (+)	Index (Antigen) test	Index manufacturer	Ref. (RT-PCR) test	Ref. manufacturer
1	Abdelrazik et al., 2020	Egypt	310	190	BIOCREDIT COVID-19 Antigen Test	RapiGen Inc., South Korea	Not available	Not available
2	Abdulrahman et al., 2020	Bahrain	4,183	734	Abbott Diagnostic GmbH, Jena, Germany	Abbott Diagnostic GmbH, Germany	TaqPath^TM^ COVID-19 Multiplex Kit	Thermo Fisher, United States
3	Albert et al., 2020	Spain	412	56	Panbio^TM^ COVID-19 Ag Rapid Test Device	Abbott Diagnostic GmbH, Jena, Germany	TaqPath^TM^ COVID-19 Multiplex Kit	Thermo Fisher, United States
4 (sx/asx)	Alemany et al., 2021	Spain	1,406	954	Panbio^TM^ COVID-19 Ag Rapid Test Device	Abbott Diagnostic GmbH, Jena, Germany	Not available	Not available
5	Aoki et al., 2020	Japan	510	26	Lumipulse^®^ SARSCoV-2 Ag kit	Fujirebio Inc., Tokyo, Japan	TaqMan^®^ Fast Virus 1-Step	Thermo Fisher Scientific, United States
6	Aoki et al., 2021	Japan	129	66	Espline^®^ SARS-CoV-2	Fujirebio Inc., Japan	TaqMan^®^ Fast Virus 1-Step	Thermo Fisher Scientific, United States
7	Beck et al., 2021	United States	346	63	Quidel Sofia^®^ SARS FIA	Quidel, San Diego, CA, United States	Cepheid Xpert^®^ Xpress	Cepheid, United States
8a	Berger et al., 2020	Switzerland	535	126	Panbio^TM^ COVID-19 Ag Rapid Test Device	Abbott Diagnostic GmbH, Germany	Roche cobas^®^ SARS-CoV-2 Assay	Roche Diagnostics, Switzerland
8b			529	193	STANDARD Q^TM^ COVID-19 Ag Test	SD Biosensor, South Korea	Roche cobas^®^ SARS-CoV-2 Assay	Roche Diagnostics, Switzerland
9 (sx/asx)	Bulilete et al., 2020	Spain	1,369	155	Panbio^TM^ COVID-19 Ag Rapid Test Device	Abbott Diagnostic GmbH, Germany	TaqPath COVID-19 Multiplex Kit	Thermo Fisher Scientific, United States
10	Cerutti et al., 2020	Italy	330	107	STANDARD Q^TM^ COVID-19 Ag Test	SD Biosensor, South Korea	Seegene Allplex^®^ 2019 n-CoV Assay	Seegene Inc., South Korea
11	Chaimayo et al., 2020	Thailand	454	63	STANDARD Q^TM^ COVID-19 Ag Test	SD Biosensor, South Korea	Seegene Allplex^®^ 2019 n-CoV Assay	Seegene Inc., South Korea
12	Ciotti et al., 2021	Italy	50	43	COVID-19 Ag Respi-Strip	Coris BioConcept, Belgium	Seegene Allplex^®^ 2019 n-CoV Assay	Seegene Inc., South Korea
13	Colavita et al., 2020	Italy	941	208	STANDARD F^TM^ COVID-19 Ag FIA	SD Biosensor, South Korea	Roche cobas^®^ SARS-CoV-2 Assay	Roche Diagnostics, Switzerland
14	Courtellemont et al., 2021	France	248	125	COVID-VIRO^®^	AAZ, France	TaqPath COVID-19 Multiplex Kit	Thermo Fisher Scientific, United States
15	Diao et al., 2020	China	251	205	SARS-CoV-2 AntigenFIC Assay (in house)	In-house	TaqMan One-Step RT-PCR Kit	Da An Gene, China
16	Drain et al., 2020	United States; United Kingdom	512	186	LumiraDx^TM^ SARS-CoV-2 Ag Test	LumiraDx, United Kingdom	Roche cobas^®^ SARS-CoV-2 Assay	Roche Diagnostics, Switzerland
17a (sx/asx)	Drevinek et al., 2020	Czech R.	591	596	Panbio^TM^ COVID-19 Ag Rapid Test Device	Abbott Diagnostic GmbH, Jena, Germany	Seegene Allplex^®^ 2019 n-CoV Assay	Seegene Inc., South Korea
17b (sx/asx)					STANDARD F^TM^ COVID-19 Ag FIA	SD Biosensor, South Korea	Seegene Allplex^®^ 2019 n-CoV Assay	Seegene Inc., South Korea
18a (sx/asx)	Favresse et al., 2021	Belgium	188	101	Biotical SARS-CoV-2 Ag card	Biotical Health, SLU, Spain	LightMix^®^	Roche Diagnostics, Switzerland
18b (sx/asx)				101	Panbio^TM^ COVID-19 Ag Rapid Test Device	Abbott Diagnostic GmbH, Germany	LightMix^®^	Roche Diagnostics, Switzerland
18c (sx/asx)				100	Coronavirus Ag Rapid Test Cassette	Healgen Scientific, LLC, United States	LightMix^®^	Roche Diagnostics, Switzerland
18d (sx/asx)				100	Roche SARS-CoV-2 Rapid Antigen Test	Roche Diagnostics, Switzerland	LightMix^®^	Roche Diagnostics, Switzerland
18e (sx/asx)				101	VITROS SARS-CoV-2 Antigen test	Ortho Clinical Diagnostics, United States	LightMix^®^	Roche Diagnostics, Switzerland
19 (sx/asx)	Fenollar et al., 2021	France	341	208	Panbio^TM^ COVID-19 Ag Rapid Test Device	Abbott Diagnostic GmbH, Germany	Vita PCR SARS-CoV-2 Assay	Credo Diagnostics, Singapore
20	Gremmels et al., 2021	Netherlands; Aruba	1,369	206	Panbio^TM^ COVID-19 Ag Rapid Test Device	Abbott Diagnostic GmbH, Germany	Seegene Allplex^®^ 2019 n-CoV Assay	Seegene Inc., South Korea
21	Hirotsu et al., 2020	Japan	313	61	Lumipulse^®^ SARS-CoV-2 Ag kit	Fujirebio Inc., Japan	TaqMan Fast Virus 1-Step Master Mix	Thermo Fisher Scientific, United States
22	Hirotsu et al., 2021	Japan	27	23	Lumipulse^®^ SARS-CoV-2 Ag kit	Fujirebio Inc., Japan	Not available	Not available
23	Hoehl et al., 2020	Germany	711	9	RIDA^®^ QUICK SARS-CoV-2 Antigen test	R-Biopharm, Darmstadt, Germany	Roche cobas^®^ SARS-CoV-2 Assay	Roche Diagnostics, Switzerland
24	Houston et al., 2021	United Kingdom	728	284	Innova SARS-CoV-2 Antigen Rapid Test	Lotus Global Company, United Kingdom	Not available	Not available
25a	Jääskeläinen et al., 2021	Finland	188	152	Quidel Sofia^®^ SARS FIA	Quidel, San Diego, CA	Not available	Not available
25b			189	162	STANDARD Q^TM^ COVID-19 Ag Test	SD Biosensor, South Korea	Not available	Not available
25c			190	156	Panbio^TM^ COVID-19 Ag Rapid Test Device	Abbott Diagnostic GmbH, Germany	Not available	Not available
26 (sx/asx)	Jakobsen et al., 2021a	Denmark	196	148	STANDARD Q^TM^ COVID-19 Ag Test	SD Biosensor, South Korea	Luna One-step RT-qPCR kit	New England Biolabs, United States
27 (sx/asx)	James et al., 2021	United States	2,339	156	BinaxNOW^TM^ COVID-19 Ag Card test kit	Abbott Diagnostics Inc., United States	PerkinElmer SARS-CoV-2 RT-PCR	PerkinElmer, Inc., United States
28	Kashiwagi et al., 2020	Japan	16	26	ESPLINE^®^ SARS-CoV-2 test	Fujirebio Inc., Japan	Not available	Not available
29a	Kohmer et al., 2021	Germany	100	76	RIDA^®^ QUICK SARS-CoV-2 Antigen test	R-Biopharm, Darmstadt, Germany	Roche cobas^®^ SARS-CoV-2 Assay	Roche Diagnostics, Switzerland
29b					Roche SARS-CoV-2 Rapid Antigen Test	Roche Diagnostics, Switzerland	Roche cobas SARS-CoV-2 Assay	Roche Diagnostics, Switzerland
29c					NADAL^®^ COVID-19 Ag Test	Nal von Minden GmbH, Germany	Roche cobas^®^ SARS-CoV-2 Assay	Roche Diagnostics, Switzerland
29d					LumiraDx^TM^ SARS-CoV-2 Ag Test	LumiraDx, United Kingdom	Roche cobas^®^ SARS-CoV-2 Assay	Roche Diagnostics, Switzerland
30a	Krüger et al., 2020	Germany; United Kingdom	417	11	COVID-19 Ag Respi-Strip	Coris BioConcept, Belgium	Roche cobas^®^ SARS-CoV-2 Assay	Roche Diagnostics, Switzerland
30b			727	18	BIOEASY^TM^ 2019-nCoV Ag Rapid Test	BIOEASY Technology, China	Roche cobas^®^ SARS-CoV-2 Assay	Roche Diagnostics, Switzerland
30c			1,267	50	STANDARD Q^TM^ COVID-19 Ag Test	SD Biosensor, South Korea	Roche cobas^®^ SARS-CoV-2 Assay	Roche Diagnostics, Switzerland
31	Krüttgen et al., 2021	Germany	150	77	Roche SARS-CoV-2 Rapid Antigen Test	Roche Diagnostics, Switzerland	Real Star SARS-CoV-2 RT PCR Kit	Altona, Germany
32	Lambert-Niclot et al., 2020	France	138	96	COVID-19 Ag Respi-Strip	Coris BioConcept, Belgium	Multiple tests used	Multiple manufacturers used
33	Linares et al., 2020	Spain	141	90	Abbott Diagnostic GmbH, Jena, Germany	Abbott Diagnostic GmbH, Germany	Seegene Allplex^®^ 2019 n-CoV Assay	Seegene Inc., South Korea
34	Lindner et al., 2020	Germany	287	41	STANDARD Q^TM^ COVID-19 Ag Test	SD Biosensor, South Korea	Roche cobas^®^ SARS-CoV-2 Assay	Roche Diagnostics, Switzerland
35	Lindner et al., 2021	Germany	146	82	STANDARD Q^TM^ COVID-19 Ag Test	SD Biosensor, South Korea	Roche cobas^®^ SARS-CoV-2 Assay	Roche Diagnostics, Switzerland
36	Liotti et al., 2020	Italy	359	107	STANDARD F^TM^ COVID-19 Ag FIA	SD Biosensor, South Korea	Multiple tests used	Multiple manufacturers used
37	Liu et al., 2021	China	99	17	In-house assay	Not applicable	Not available	Not available
38a	Mak et al., 2020	China	140	143	COVID-19 Ag Respi-Strip	Coris BioConcept, Belgium	Not available	Not available
38b					NADAL^®^ COVID-19 Ag Test	Nal von Minden GmbH, Germany	Not available	Not available
38c					STANDARD Q^TM^ COVID-19 Ag Test	SD Biosensor, South Korea	Not available	Not available
39	Mak et al., 2021	China	105	108	Abbott Diagnostic GmbH, Jena, Germany	Abbott Diagnostic GmbH, Germany	Not available	Not available
40 (sx/asx)	Masiá et al., 2020	Spain	913	40	Abbott Diagnostic GmbH, Jena, Germany	Abbott Diagnostic GmbH, Germany	Roche cobas^®^ SARS-CoV-2 Assay	Roche Diagnostics, Switzerland
41	Menchinelli et al., 2021	Italy	594	196	Lumipulse^®^ SARS-CoV-2 Ag kit	Fujirebio Inc., Japan	Seegene Allplex^®^ 2019 n-CoV Assay	Seegene Inc., South Korea
42	Merino-Amador et al., 2020	Spain	958	361	Abbott Diagnostic GmbH, Jena, Germany	Abbott Diagnostic GmbH, Germany	Not available	Not available
43	Möckel et al., 2021	Spain	473	115	Roche SARS-CoV-2 Rapid Antigen Test	Roche Diagnostics, Switzerland	Roche cobas^®^ SARS-CoV-2 Assay	Roche Diagnostics, Switzerland
44	Nalumansi et al., 2020	Uganda	262	94	STANDARD Q^TM^ COVID-19 Ag Test	SD Biosensor, South Korea	Not available	Not available
45	Ngo Nsoga et al., 2021	Switzerland	402	169	Abbott Diagnostic GmbH, Jena, Germany	Abbott Diagnostic GmbH, Germany	Roche cobas^®^ SARS-CoV-2 Assay	Roche Diagnostics, Switzerland
46	Okoye et al., 2021	United States	2,638	46	BinaxNOW^TM^ COVID-19 Ag Card test kit	Abbott Diagnostics, Inc., United States	TaqPath COVID-19 Multiplex Kit	Thermo Fisher Scientific, United States
47a	Osterman et al., 2021	Germany	445	192	STANDARD F^TM^ COVID-19 Ag FIA	SD Biosensor, South Korea	RealAccurate^®^ Quadruplex SARS CoV-2 PCR Kit	PathoFinder^®^, Netherlands
47b				259	Roche SARS-CoV-2 Rapid Antigen Test	Roche Diagnostics, Switzerland	RealAccurate^®^ Quadruplex SARS CoV-2 PCR Kit	PathoFinder^®^, Netherlands
48	Pekosz et al., 2021 ^a^	United States	251	38	BD Veritor^TM^ SARS-CoV-2 Rapid Antigen test	Becton, Dickinson and Company, United States	Lyra^®^ RT-PCR Assay	Quidel Corporation, United States
49a	Peto, 2021	United Kingdom	940	198	Innova SARS-CoV-2 Antigen Rapid Test	Lotus Global Company, United Kingdom	Not available	Not available
49b				100	Abbott Diagnostic GmbH, Jena, Germany	Abbott Diagnostic GmbH, Germany	Not available	Not available
50 (sx/asx)	Pilarowski et al., 2020a	United States	878	131	BinaxNOW^TM^ COVID-19 Ag Card test kit	Abbott Diagnostics Scarborough, Inc., United States	Not available	Not available
51 (sx/asx)	Pilarowski et al., 2020b	United States	3,302	134	BinaxNOW^TM^ COVID-19 Ag Card test kit	Abbott Diagnostics Scarborough, Inc., United States	Renegade XP^TM^	RenegadeBio, United States
52	Pollock et al., 2021b	United States	226	139	S-PLEX^®^ SARS-CoV-2 Assay	Meso Scale Discovery, United States	Not available	Not available
53 (sx/asx)	Pollock et al., 2021a	United States	2,308	295	BinaxNOW^TM^ COVID-19 Ag Card test kit	Abbott Diagnostics Scarborough, Inc., United States	CRSP SARS-CoV-2 RT-PCR	Harvard University, United States
54	Porte et al., 2020	Chile	127	83	BIOEASY^TM^ 2019-nCoV Ag Rapid Test	BIOEASY Technology, China	Genesig^®^ Real-Time PCR assay	Primerdesign Ltd., United Kingdom
55 (sx/asx)	Pray et al., 2021	United States	1,098	59	Quidel Sofia^®^ SARS FIA	Quidel, San Diego, CA	Multiple tests used	Multiple manufacturers used
56 (sx/asx)	Prince-Guerra et al., 2021	United States	3,419	226	BinaxNOW^TM^ COVID-19 Ag Card test kit	Abbott Diagnostics, Inc., United States	Multiple tests used	Multiple manufacturers used
57	Rastawicki et al., 2021	Poland	167	38	PCL COVID−19 Ag rapid immunoassay	PLC, South Korea	Multiple tests used	Multiple manufacturers used
58a	Schwob et al., 2020	Switzerland	928	113	STANDARD Q^TM^ COVID-19 Ag Test	SD Biosensor, South Korea	Not available	Not available
58b				123	Abbott Diagnostic GmbH, Jena, Germany	Abbott Diagnostic GmbH, Germany	Not available	Not available
58c				139	COVID-VIRO^®^	AAZ, France	Not available	Not available
59 (sx/asx)	Scohy et al., 2020	Belgium	148	93	COVID-19 Ag Respi-Strip	Coris BioConcept, Belgium	Genesig^®^ Real-Time PCR assay	Primerdesign Ltd., United Kingdom
60	Stokes et al., 2021	Canada	145	140	Abbott Diagnostic GmbH, Jena, Germany	Abbott Diagnostic GmbH, Germany	Not available	Not available
61	Strömer et al., 2020	Germany	134	126	NADAL^®^ COVID-19 Ag Test	Nal von Minden GmbH, Germany	Not available	Not available
62 (sx/asx)	Takeuchi et al., 2021	Japan	771	74	Quick Navi^TM^-COVID 19 Ag Rapid Test	Otsuka Pharmaceutical Co., Ltd., Japan	Not available	Not available
63	Toptan et al., 2020	Germany	67	61	RIDA^®^ QUICK SARS-CoV-2 Antigen test	R-Biopharm, Darmstadt, Germany	Roche cobas^®^ SARS-CoV-2 Assay	Roche Diagnostics, Switzerland
64	Torres et al., 2021	Spain	634	80	Abbott Diagnostic GmbH, Jena, Germany	Abbott Diagnostic GmbH, Germany	TaqPath COVID-19 Multiplex Kit	Thermo Fisher Scientific, United States
65 (sx/asx)	Turcato et al., 2020	Italy	3,410	227	STANDARD Q^TM^ COVID-19 Ag Test	SD Biosensor, South Korea	Not available	Not available
66	Van der Moeren et al., 2020	Netherlands	352	125	BD Veritor^TM^ SARS−CoV−2 Rapid Antigen test	Becton, Dickinson and Company, United States	Roche cobas^®^ SARS-CoV-2 Assay	Roche Diagnostics, Switzerland
67	Villaverde et al., 2021	Spain	1,620	79	Abbott Diagnostic GmbH, Jena, Germany	Abbott Diagnostic GmbH, Germany	Not available	Not available
68a	Weitzel et al., 2020	Chile	111	81	Biocredit COVID-19 Antigen Test	RapiGen Inc., South Korea	Genesig^®^ Real-Time PCR assay	Primerdesign Ltd., United Kingdom
68b				80	Huaketai New Coronavirus	Savant Biotechnology Co., Ltd., China	Genesig^®^ Real-Time PCR assay	Primerdesign Ltd., United Kingdom
68c				82	BIOEASY 2019-nCoV Ag Rapid Test	BIOEASY^TM^ Technology, China	Genesig^®^ Real-Time PCR assay	Primerdesign Ltd., United Kingdom
69	Yamamoto et al., 2021	Japan	608	130	In-house assay	Not applicable	Not available	Not available
70	Young et al., 2020 ^b^	United States	226	32	BD Veritor^TM^ SARS−CoV−2 Rapid Antigen test	Becton, Dickinson and Company, United States	Lyra^®^ RT-PCR Assay	Quidel Corporation, United States
71	Abbott Diagnostics Scarborough Inc, 2020a	United States	460	120	BinaxNOW^TM^ COVID-19 Ag Card test kit	Abbott Diagnostics, Inc., United States	Roche cobas^®^ SARS-CoV-2 Assay	Roche Diagnostics, Switzerland
72	Abbott Diagnostics Scarborough Inc, 2020b	United States	53	27	BinaxNOW^TM^ COVID-19 Ag Card test kit	Abbott Diagnostics, Inc., United States	Roche cobas^®^ SARS-CoV-2 Assay	Roche Diagnostics, Switzerland
73	Access Bio, 2020	United States	126	46	CareStart^TM^ COVID-19 Rapid Test	Access Bio, Inc., United States	Not available	Not available
74	Ellume Limited, 2020	United States	198	40	Ellume COVID-19 Home Test	Ellume Limited, United States	Not available	Not available
75	Luminostics Inc, 2020	United States	166	35	Clip COVID-Rapid Antigen Test	Luminostics, Inc., United States	Not available	Not available
76	LumiraDx UK Ltd, 2020	United Kingdom	257	86	LumiraDx^TM^ SARS-CoV-2 Ag Test	LumiraDx, United Kingdom	Roche cobas^®^ SARS-CoV-2 Assay	Roche Diagnostics, Switzerland
77	Quidel Corporation, 2020a	United States	156	61	QuickVue^®^ SARS Antigen Test	Quidel Corporation, United States	Not available	Not available
78	Celltrion, 2020	United States	72	39	Sampinute^TM^ COVID-19 Antigen MIA	Celltrion, United States	Not available	Not available
79	Quidel Corporation, 2020b	United States	209	33	Quidel Sofia^®^ SARS FIA	Quidel, United States	Lyra^®^ RT-PCR Assay	Quidel Corporation, United States
80	Quidel Corporation, 2020c	United States	165	45	Quidel Sofia^®^ SARS FIA	Quidel, United States	Lyra^®^ RT-PCR Assay	Quidel Corporation, United States
81	BD, 2020a	United States	226	32	BD Veritor^TM^ SARS−CoV−2 Rapid Antigen test	Becton, Dickinson and Company, United States	Lyra^TM^ RT-PCR Assay	Quidel Corporation, United States
82	BD, 2020b	EU	251	38	BD Veritor^TM^ SARS−CoV−2 Rapid Antigen test	Becton, Dickinson and Company, United States	Lyra^®^ RT-PCR Assay	Quidel Corporation, United States
83	BD, 2020c	United States	115	61	BD Veritor^TM^ SARS−CoV−2 Rapid Antigen test	Becton, Dickinson and Company, United States	Lyra^®^ RT-PCR Assay	Quidel Corporation, United States

*sx/ask, symptomatic/asymptomatic; SARS-CoV-2, severe acute respiratory syndrome coronavirus-2.*

*EU, European Union; IFU, manufacturer instructions for use; RFID, reference ID; RT-PCR, reverse transcription polymerase chain reaction.*

*^*a*^Pekosz 2021 was only used as a data source for sensitivity analysis with Ct score stratification and for sensitivity analysis using cell culture as a reference.*

*^*b*^Young 2020 was only used as a data source for sensitivity analysis for Ct score stratification.*

**FIGURE 3:**
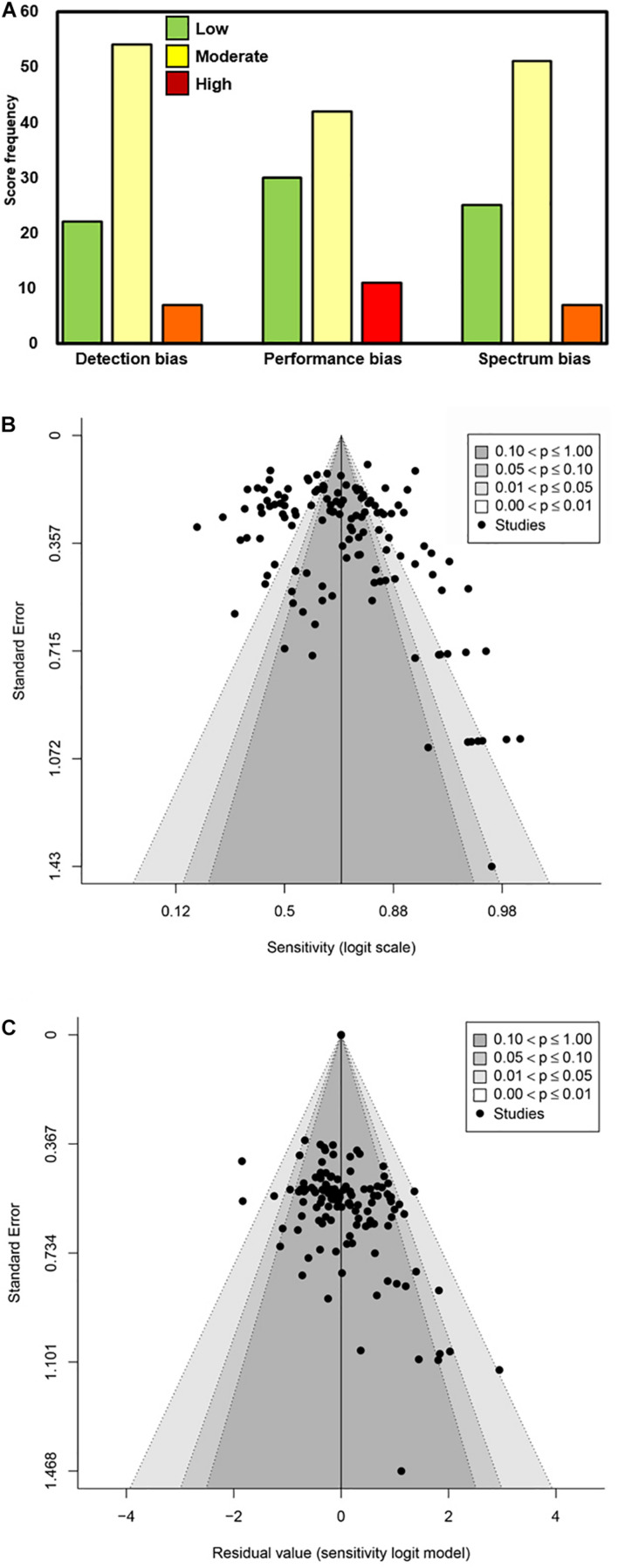
Determination of bias associated with the source articles/documents included in this meta-analysis. **(A)** Scoring as Low, Moderate, and High was performed for Detection bias, Performance bias, and Spectrum bias associated with each data source included. The frequency of the scores is plotted along the *Y*-axis. **(B,C)** Funnel plots of logit-transformed sensitivity in a model without any moderators **(B)** and with moderators included **(C)**.

A modified Newcastle–Ottawa Scale was used to rate biases of detection, performance, and participant selection (spectrum bias; Figure 3). The majority of studies were associated with low or moderate bias of detection (89.1%; 74/83), bias of performance (85.5%; 71/83), and spectrum bias (87.9%; 73/83). All included articles/sources had acceptable reference standards (that was an inclusion criterion), appropriate delay between index and reference testing, and no incorporation bias between the index and reference tests. The two most common weaknesses associated with study design for the included articles/sources were improper blinding and spectrum bias associated with participant enrollment (Lijmer et al., 1999). Across the six primary factors (viral load, symptomatic vs. asymptomatic, DSO, anatomical collection site, storage condition, and analytical sensitivity of the reference RT-qPCR assay) analyzed here, the quality of evidence was largely a mixture of high and moderate (Table 2).

**TABLE 2:** Overall quality of evidence for outcomes^a^ (modified GRADE; Schunemann et al., 2013).

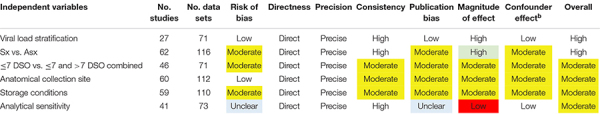

*GRADE, Grading of Recommendations, Assessment, Development and Evaluation; Sx, symptomatic; Asx, asymptomatic; DSO, days from symptom onset.*

*^*a*^Each independent variable (e.g., Viral Load Stratification), was rated according to seven quality domains, with an overall quality rating in the last column. The overall quality rating was established based on the overall number of green (indicating high quality), yellow (indicating moderate quality), and red (indicating low quality) domains; blue shading indicates that the quality rating for that domain was unclear. For example, low Publication Bias and high Consistency are both rated as high quality, whereas a low Magnitude of Effect resulted in a low quality rating. Please see the Methods and Materials section for further description of the overall quality ranking.*

*^*b*^Counfounder effect characterizes the degree to which all plausible confounders would tend to increase confidence in the estimated effect.*

**FIGURE 4:**
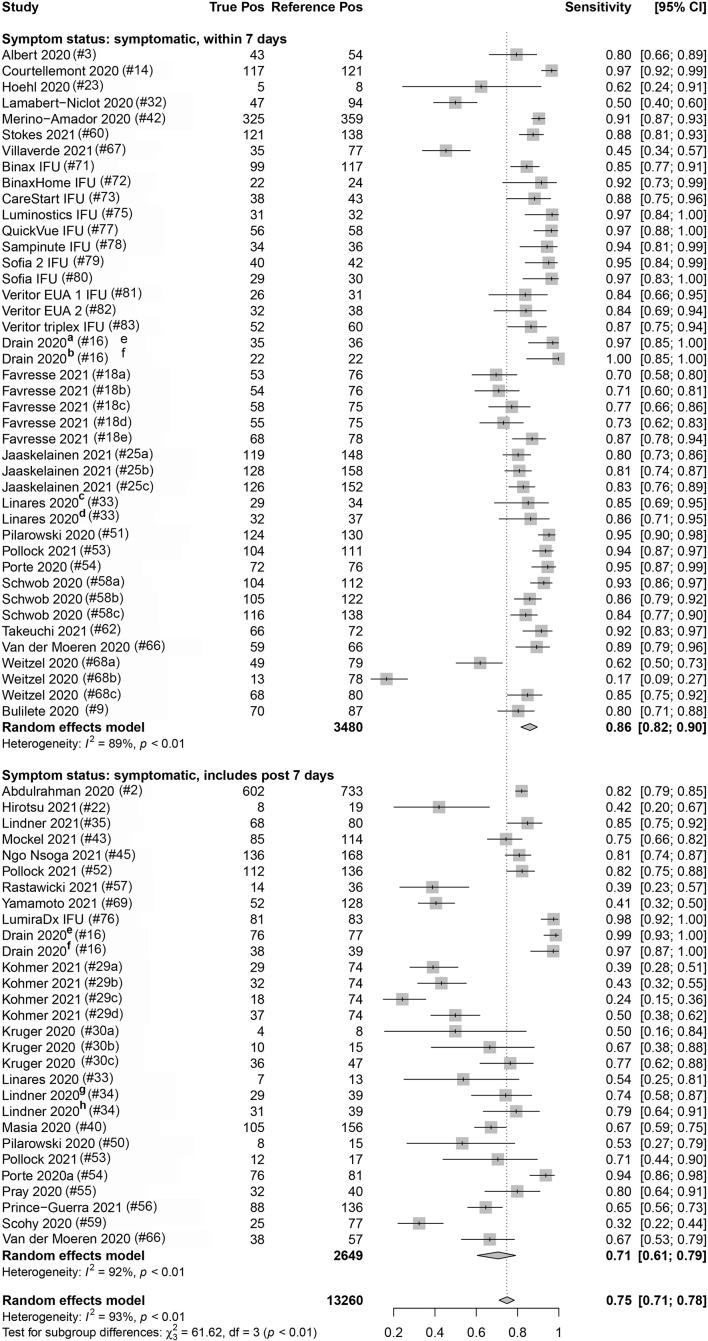
Forest plots containing calculated sensitivity values for index [severe acute respiratory syndrome coronavirus-2 (SARS-CoV-2) antigen test] testing compared to reference [SARS-CoV-2 quantitative reverse transcription polymerase chain reaction (RT-qPCR assay)] test. Data are stratified by days from symptom onset.

Eighty-three percent (112/135) and 16.3% (22/135) of the data sets provided data from COVID-19 symptomatic and asymptomatic individuals, respectively. The index test sensitivity point estimate (with 95% CI) for the symptomatic group [80.1% (95% CI: 76.0, 83.7); reference positive *n* = 9,351] was significantly greater (*p*-value < 0.001) than that for index test sensitivity for the asymptomatic group [54.8% (95% CI: 48.6, 60.8); reference positive *n* = 1,723]. Of the 112 symptomatic data sets, 37.5% (42) included participants that were ≤7 DSO, and 25.9% (29) included individuals that were a mix of ≤7 DSO and >7 DSO; 36.6% (41) had a DSO status that was unknown. A significant difference (*p*-value = 0.001) was observed when studies reporting on symptomatic individuals were subgrouped by DSO; a sensitivity point estimate of 86.2% (95% CI: 81.8, 89.7) for the ≤7 DSO subgroup (reference positive *n* = 3,480) compared to 70.8% (95% CI: 60.7, 79.2) for the group including both ≤7 DSO and >7 DSO (reference positive *n* = 2,649; Figure 4 and Table 3).

**TABLE 3:** Diagnostic performance (sensitivity) for antigen testing, with RT-qPCR as reference, stratified by different population and experimental factors.

Variable category	Variable group	No. data sets	Total reference (+)	Sensitivity (%)	95% CI	Univariate^a^	Multivariate
RT-qPCR Ct value	≥10^5^ cpm	18	1,278	93.8	[87.1, 97.1]	*p* < 0.001	n/a
	<10^5^cpm	18	781	28.6	[16.2, 45.3]		
	Ct value ≤ 25	16	897	96.4	[94.3, 97.7]	*p* < 0.001	n/a
	Ct value > 25	16	673	44.9	[33.0, 57.4]		
	Ct value ≤ 30	37	2,536	89.5	[85.3, 92.5]	*p* < 0.001	n/a
	Ct value > 30	37	679	18.7	[12.9, 26.3]		
Symptom status	Symptomatic	95	9,351	80.1	[76.0, 83.7]	*p* < 0.001	*p* < 0.001
	Asymptomatic	23	1,723	54.8	[48.6, 60.8]		
	Missing information	15	2,186	65.3	[56.4, 73.3]		
	≤7 DSO	42	3,480	86.2	[81.8, 89.7]		
	≤7 DSO + > 7 DSO	29	2,649	70.8	[60.7, 79.2]		
Anatomical site (ref.)	Nasal swab	25	1,654	82.7	[74.7, 88.5]	*p* = 0.037	*p* = 0.023
	NPS	97	10,607	73.1	[68.5, 77.2]		
	Missing	11	999	71.6	[55.7, 83.6]		
Specimens storage (index)	Fresh	101	9,666	75.3	[70.8, 79.3]	*p* = 0.375	
	Frozen	23	3,307	70.9	[61.0, 79.1]		
	Missing	9	287	81.5	[69.3, 89.5]		
Analytical sensitivity (Ref.)	High (≤500 cpm)	53	4,531	74.2	[66.9, 80.4]	*p* = 0.650	
	Low (>500 cpm)	22	2,582	76.8	[67.2, 84.2]		
	Missing	58	6,147	75.0	[69.8, 79.5]		
Study spectrum bias	High/Moderate	52	5,524	81.4	[75.5, 86.1]	*p* = 0.004	*p* = 0.757
	Low	81	5,871	70.4	[65.4, 74.9]		
All sources combined	n/a	133	13,260	75.0	[71.0, 78.0]		

*CI, confidence interval; DSO, days from symptom onset; Ct, cycle threshold; cpm, genomic copies/ml; NPS, nasopharyngeal swab; and RT-qPCR, quantitative reverse transcription polymerase chain reaction.*

*^*a*^*Q-*test *p*-value for heterogeneity among subgroups calculated from random-effects meta-analysis.*

**FIGURE 5:**
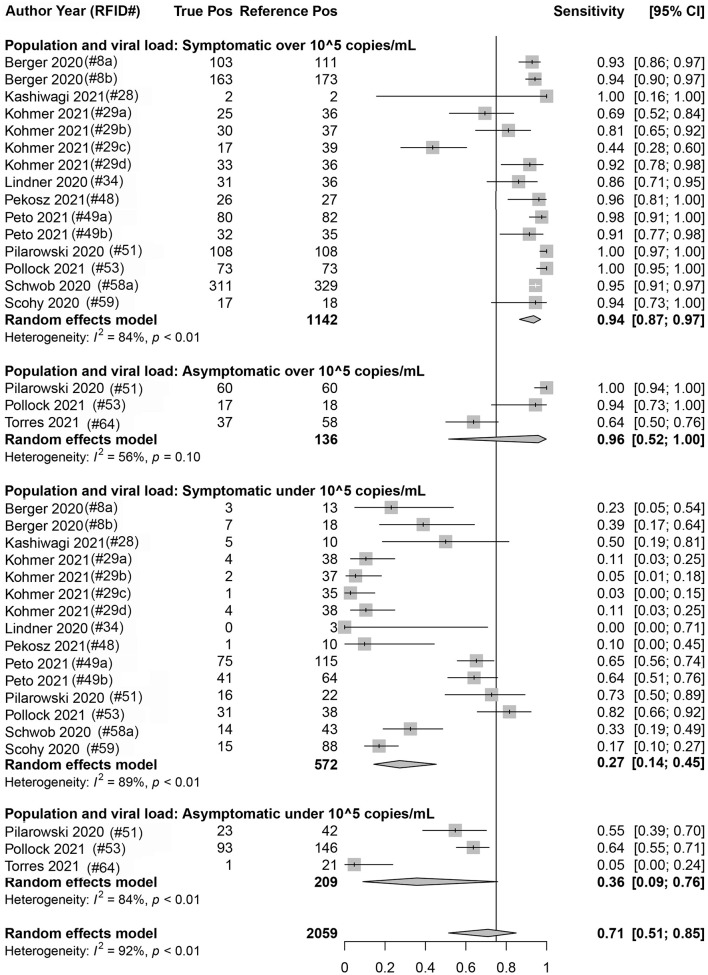
Forest plots containing calculated sensitivity values for index [severe acute respiratory syndrome coronavirus-2 (SARS-CoV-2) antigen test] testing compared to reference [SARS-CoV-2 quantitative reverse transcription polymerase chain reaction (RT-qPCR assay)] test. Data are stratified by viral load [genomic copies/ml (cpm)] and by symptomatic or asymptomatic status.

Eighteen data sets reported true positives and false negatives by viral load in the specimen; 37 and 16 data sets reported values by a Ct value of 30 and 25, respectively, for the RT-qPCR (reference) assay. When data were stratified by ≥1 × 10^5^ cpm (*n* = 1,278 reference positive results) vs. <1 × 10^5^ cpm (*n* = 781 reference positive results), a significant difference was observed (*p* < 0.001) between the sensitivity point estimates [93.8% (95% CI: 87.1, 97.1) and 28.6% (95% CI: 16.2, 45.3), respectively]. Similar findings were associated with studies that were stratified by a Ct value of ≤30 (*n* = 2,536 reference positive results) and >30 (*n* = 679 reference positive results) [89.5% (95% CI: 85.3, 92.5) and 18.7% (95% CI: 12.9, 26.3), respectively] and those that were stratified by a Ct value of ≤25 (*n* = 897 reference positive results) and >25 (*n* = 673 reference positive results) [96.4% (95% CI: 94.3, 97.7) and 44.9% (95% CI: 33.0, 57.4), respectively]. Within a given viral load category, there were no statistical differences between studies performed with symptomatic subjects vs. studies performed with asymptomatic subjects; this was true regardless of the exact definition of viral load category: Ct of 25, Ct of 30, or cpm of 10^5^ (Figure 5 and Table 3).

True positives and false negatives by anatomic collection site were obtained from 97 data sets that included reference nasopharyngeal specimens and from 25 data sets that included nasal reference specimens. Antigen testing was usually paired from the same specimen type, only six data sets being non-paired (antigen nasal, reference nasopharyngeal) specimens. When analysis was performed on data stratified by anatomic collection site of the reference specimen, antigen test sensitivity was higher with a nasal specimen [82.7% (95% CI: 74.7, 88.5, *p* = 0.037); reference positive *n* = 1,654] compared to a nasopharyngeal specimen [73.1% (95% CI: 68.5, 77.2); reference positive *n* = 10,607] (Table 3).

Storage condition of the collected specimens was also analyzed as one factor that could affect antigen test sensitivity. True positives and false negatives by test storage condition of the specimen that underwent antigen testing were obtained from 133 data sets. When analysis was focused on storage condition for index testing, antigen test sensitivity was 75.3% (95% CI: 70.8, 79.3; reference positive *n* = 9,666) for fresh specimens and 70.9% (95% CI: 61.0, 79.1; reference positive *n* = 3,307) for frozen specimens. This observed difference was not, however, statistically significant (*p* = 0.375; Table 3).

Analytical sensitivity of the reference method (RT-qPCR) was determined using the manufacturer’s IFU when it was identified in the source documents and used to stratify true-positive and false-negative results associated with SARS-CoV-2 antigen testing. The LOD threshold for low and high analytical sensitivity was 500 cpm, which was the median (mean = 582) LOD value for the analytical sensitivity from all of the reference methods included in this subanalysis. Sensitivity values for antigen testing when stratified by high (reference positive *n* = 4,468) and low (reference positive *n* = 2,645) analytically sensitive reference methods were similar: 74.2% (95% CI: 66.9, 80.4) and 76.8% (95%CI: 67.2, 84.2), respectively (Table 3).

Manufacturer (Supplementary Table 1) and study spectrum bias were also significant factors in subgroup meta-analyses; higher sensitivity was reported in studies with large/moderate spectrum bias (Table 3). A mixed-effects meta-regression model with moderators including symptom status, anatomical collection site, study selection/spectrum bias, and manufacturer was fit to the studies. All factors remained significant in the multivariate analysis, except study spectrum bias (multivariate *p* = 0.757). The moderators accounted for 72% of study heterogeneity (model *R*^2^ = 0.722). Visual inspection of unadjusted and multivariate-adjusted funnel plots for effect estimates from individual sources against study size was performed (Figure 3). The funnel plot asymmetry revealed possible reporting/publication bias reflecting fewer studies than expected that could be characterized by a small group number and a low sensitivity estimate for the index. Overall, study heterogeneity could largely be accounted for by the independent variables identified through subgroup analysis in this study.

### Culture as the Reference

**FIGURE 6:**
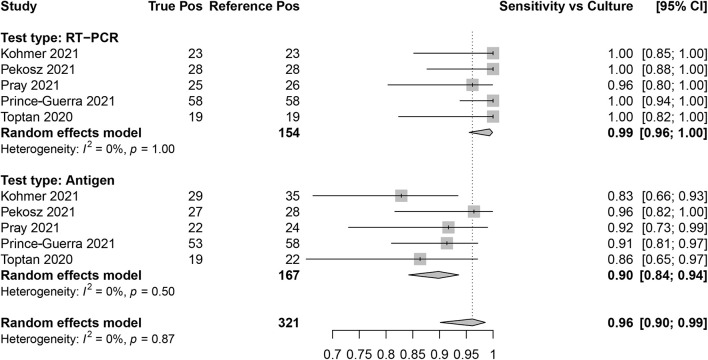
Forest plots containing calculated sensitivity values for index [severe acute respiratory syndrome coronavirus-2 (SARS-CoV-2) antigen test and SARS-CoV-2 quantitative reverse transcription polymerase chain reaction (RT-qPCR assay)] testing compared to reference (SARS-CoV-2 viral culture) test.

Sensitivity for SARS-CoV-2 antigen and RT-qPCR assays was determined as compared with SARS-CoV-2 viral culture as the reference method. There were five data sets that contained RT-qPCR (reference positive *n* = 154) and antigen test (reference positive *n* = 167) results. The overall sensitivity for RT-qPCR was 99.0% (95% CI: 96.0, 100) and for antigen testing was 90.0% (95% CI: 84.0, 94.0; Figure 6).

### Specificity

Raw data for false-positive and true-negative rates were extracted from 63 of 81 of the included studies; the overall specificity across the included studies was 99.4% (95% CI: 99.3, 99.4) for antigen testing compared to RT-qPCR as the reference.

## Discussion

The positive percent agreement point estimate (sensitivity) for antigen testing, spanning the entire 135 data sets included here, was 75.0% (95% CI: 71.0, 79.0). We found that factors including specimen viral load, symptom presence, DSO, anatomical collection site, and the storage conditions for specimen collection could all affect the measured performance of SARS-CoV-2 antigen tests (Supplementary Table 1 and Supplementary Figure 1). In addition, our meta-analysis revealed that antigen test sensitivity [96.0% (95% CI: 90.0, 99.0)] was highest in SARS-CoV-2-positive individuals with an increased likelihood of being infectious at the time of testing (e.g., culture positive; Pekosz et al., 2021). Although specificity data were not extracted for every study included in this meta-analysis, SARS-CoV-2 antigen testing had high specificity as published previously (Brümmer et al., 2021). Experimental factors such as anatomical collection site, specimen storage conditions, analytical sensitivity of reference, and composition of the study population with respect to symptomology all varied across the field of studies included here.

This meta-analysis adds to the conclusions of others that viral load is clearly the most important factor that influences sensitivity for SARS-CoV-2 antigen testing. Two related meta-analyses have been published to date. The first by Dinnes et al. (2020) had the following key differences: (1) Dinnes et al. (2020) utilized numerous categories of point-of-care tests beyond antigen-based testing; (2) Dinnes et al. (2020) included five articles for antigen testing in their work; and (3) Dinnes et al. (2020) did not stratify the meta-analysis results for antigen testing by study design/viral load as is performed in this work. The second by Brümmer et al. (2021) had the following key differences: (1) Brümmer et al. (2021) focused on commercial rapid antigen tests; (2) Brümmer et al. (2021) only included 48 articles for antigen testing in their work; and (3) Brümmer et al. (2021) did not stratify the meta-analysis results for antigen test performance by study design characteristics as is performed in this work.

Test sensitivity was stratified by RT-qPCR Ct value (using both 25 cycles and 30 cycles as the cutoff); an inverse relationship was shown between Ct value and SARS-CoV-2 antigen test sensitivity. Both the ≤25 Ct group and the ≤30 Ct group had significantly higher sensitivities than their >25 Ct and >30 Ct counterparts, respectively, regardless of subjects’ symptom status. These results are consistent with those from previous studies (COVID-19 Scientific Advisory Group Rapid Evidence Report, 2020; Dinnes et al., 2020; Brümmer et al., 2021). However, Ct value has been shown by different groups to have a low correlation between different RT-qPCR assays and platforms (Ransom et al., 2020; Rhoads et al., 2020). RT-qPCR assays have different analytical sensitivities; a universal Ct value reference has not been established that can be used to define the optimal sensitivity/specificity characteristics for antigen testing. In addition to stratification by Ct value, analysis was also performed for SARS-CoV-2 antigen testing sensitivity by absolute viral load (using 1 × 10^5^ as the cutoff). When data were analyzed using this strategy, similar results were observed as for stratification by Ct value. The viral load threshold utilized here was determined by a consensus value that appeared with regular frequency from the source articles and represented a viral threshold that consistently delineated a zone across which the false-positive rate increased for most antigen tests. It is generally accepted that viral loads of less than 1 × 10^5^ cpm correlate with non-culture-positive levels. However, whether 1 × 10^5^ cpm is the most accurate threshold by which to measure antigen test performance is still a topic for debate. Some studies suggest that viral loads closer to 1 × 10^6^ cpm might be a more appropriate threshold, which would act to minimize false-positive rates (Berger et al., 2020; Larremore et al., 2020; La Scola et al., 2020; Quicke et al., 2020; van Kampen et al., 2020; Wolfel et al., 2020).

Several factors identified here that affect SARS-CoV-2 antigen test performance have been identified in previous studies to affect specimen viral load. For example, a significant difference in sensitivity for detection was noted here between symptomatic and asymptomatic individuals. However, stratification in both symptomatic and asymptomatic specimen groups by high viral load has a similar effect of increasing test sensitivity. Our data show that 84% of SARS-CoV-2-positive specimens from symptomatic individuals corresponded to a high viral load, whereas only 56% of SARS-CoV-2-positive specimens from asymptomatic individuals qualified as high viral load in this analysis. This bias may be due to the difficulty of estimating the timing of peak viral load in asymptomatic individuals when attempting to compare the natural history of viral load trajectory in symptomatic vs. asymptomatic individuals (Smith et al., 2021). Nevertheless, the presence of symptoms probably overlaps with a higher specimen viral load, which subsequently affects the antigen test sensitivity. Anatomical collection type of the index and/or reference test method can affect the measured sensitivity estimates of antigen testing during a clinical trial, also through a mechanism that involves increased/decreased viral load on the specimen swab. Evidence suggests that viral loads may be higher with nasopharyngeal than with nasal collection (Pinninti et al., 2020). This difference may explain why measured antigen assay performance appears to be higher in studies that use a nasal RT-qPCR reference method.

Another factor identified here as potentially influencing measured antigen assay sensitivity was specimen storage, particularly with regard to the use of fresh vs. frozen (i.e., “banked”) specimens. It is likely that protein antigen may, as the result of freeze/thawing, experience some degree of structural damage potentially leading to loss of epitope availability or a reduction in the affinity of epitope/paratope binding. Ninety-six data sets involved fresh specimens for antigen testing, and 23 data sets included freeze/thawed specimens for antigen testing. Although no statistically significant difference was detected between sensitivities for antigen test conducted on fresh vs. frozen specimens, possibly due to the low data set group number in the frozen antigen group, a trend toward lower sensitivity was observed for tests performed on frozen specimens [75.3% (95% CI: 70.8, 79.3) for fresh vs. 70.9% (95% CI: 61.0, 79.1) for frozen]. In contrast, no similar trend was observed for specimen storage condition related to RT-qPCR testing [75.4% (95% CI: 70.6, 79.6) for fresh vs. 77.7% (95% CI: 69.3, 84.3) for frozen]. Additional results from in-house (i.e., a BD-IDS laboratory) testing with two different EUA authorized antigen assays demonstrate reduced immunoassay band intensity following freeze–thaw cycles, thus further supporting the findings from the meta-analysis that a freeze–thaw cycle could reduce analytical sensitivity for SARS-CoV-2 antigen testing (Supplementary Figure 2).

The analytical sensitivity associated with the reference RT-qPCR assay was also investigated here as a possible variable that could affect the false-negative rate of SARS-CoV-2 antigen testing. We hypothesized that relatively high analytical sensitivity for the reference RT-qPCR assay would impose a detection bias and result in decreased clinical sensitivity due to increased false negatives occurring near the RT-qPCR LOD. However, stratification by reference analytical sensitivity resulted in no difference in SARS-CoV-2 antigen test clinical sensitivity. It is likely that the analytical sensitivity of RT-qPCR, regardless of the manufacturer, is high enough that even relatively low sensitivity RT-PCR assays are still well below the corresponding LOD for antigen testing. On the other hand, some manufacturers evaluate antigen test performance in a manner that involves sensitivity above and below a set Ct value. It is possible that analysis involving stratification by RT-qPCR analytical sensitivity could reveal differences in antigen test performance if all antigen test performances were determined in a similar manner that involves predetermined high/low viral load categories.

Several population- and study design-specific factors were identified to be associated with higher measured assay sensitivity likely due to the association with higher viral loads. This meta-analysis demonstrates that these factors exist in various combinations across studies in an inconsistent way, thus making comparisons of assay performance across these studies impossible. The lack of consistency across study designs makes it very difficult to compare point estimates between antigen tests to judge their relative clinical efficacy. The introduction of different forms of bias into the study design, and during study conduct, could explain why discrepancies have been noted, for example, between sensitivity values listed in manufacturers’ IFUs and those obtained during independent evaluation of the same antigen test. Ultimately, direct comparison between antigen tests should be the most reliable approach for obtaining relative performance characteristics with any certainty. Here, we stratified SARS-CoV-2 antigen test sensitivity by spectrum bias associated with each of the data sources. We found that those studies rated with higher spectrum bias also had higher antigen test sensitivities. In addition, the funnel plot analysis that was performed for this meta-analysis shows obvious publication bias, which implicated a lack of publication of studies with low study group number and low sensitivity.

Clinical trials and studies involving diagnostics are vulnerable to the introduction of bias, which can alter test performance results and obstruct an accurate interpretation of clinical efficacy or safety. For example, antigen testing appears to have a higher sensitivity when compared to SARS-CoV-2 viral culture as the reference than when compared to RT-PCR. However, these two reference methods measure different targets: RNA only vs. infectious virus. Therefore, their use as a reference method should be intended to answer different scientific questions rather than artificially inflating apparent sensitivity point estimates. If the intent of a diagnostic test is determining increased risk of infectiousness through the presence of replicating virus, the high analytical sensitivity of RT-qPCR, which cannot distinguish RNA fragments from infectious virus, renders this diagnostic approach vulnerable to the generation of false-positive results, particularly at later time points following symptom onset. At time points beyond 1 week from symptom onset, a positive RT-qPCR result more likely indicates that an individual has been infected but is no longer contagious and cannot spread infectious virus. This is especially true for those with a SARS-CoV-2-negative cell culture result. Previous reports have shown that performance values for rapid antigen tests and SARS-CoV-2 viral culture exhibit better agreement than do results from RT-qPCR compared to viral culture in symptomatic individuals, thus making it a good test to identify individuals who are likely to be shedding infectious virus and therefore have potential to transmit SARS-CoV-2 (Pekosz et al., 2021). With this current analysis, we further show that antigen testing is also able to reliably identify asymptomatic individuals with viral load indicative of shedding infectious virus (1 × 10^5^ cpm and/or a Ct score ≤30).

This work focused on factors that can affect antigen test sensitivity for detection of SARS-CoV-2. RT-qPCR testing and other forms of testing (such as molecular point-of-care assays or serological testing) have been characterized and described as diagnostic approaches for SARS-CoV-2 elsewhere (Carter et al., 2020). Several assay characteristics, including time to result (Peeling et al., 2021), analytical sensitivity (Mak et al., 2020), cost per test (Neilan et al., 2020; Kepczynski et al., 2021; Love et al., 2021; Jakobsen et al., 2021b), infrastructure requirements for testing (Augustin et al., 2020), and volume of testing (Carter et al., 2020), need to be considered before determining the most appropriate testing strategy. As the priorities for specific test characteristics differ between testing sites, so does the overall value of a given diagnostic test.

## Limitations

This study has some limitations. First, it was difficult to obtain reliable information across the sources, in a consistent manner, about disease severity in order to perform a meta-analysis on this aspect of COVID-19 diagnostics. Additionally, the studies included in this meta-analysis did not contain sufficient information to explore the potential effect of factors previously demonstrated to be associated with higher viral loads such as disease severity and community prevalence.

## Conclusion

In addition to viral load, several factors including symptom status, anatomical collection site, and spectrum bias all influenced the sensitivity for SARS-CoV-2 detection by antigen-based testing. This heterogeneity of factors found to influence measured assay sensitivity, across studies, precludes comparison of assay sensitivity from one study to another. Future consideration regarding standardization of these factors for antigen assay performance studies is warranted in order to aid in results interpretation and relative performance assessment.

## Data Availability Statement

The original contributions presented in the study are included in the article/Supplementary Material, further inquiries can be directed to the corresponding author/s.

## Author Contributions

All authors contributed to the interpretation of the data, critically revised the manuscript for important intellectual content, approved the final version to be published, and agree to be accountable for all aspects of the work.

## Author Disclaimer

The views expressed in this article are those of the authors and do not necessarily represent the views of the National Institute of Biomedical Imaging and Bioengineering; the National Heart, Lung, and Blood Institute; the National Institutes of Health; or the United States Department of Health and Human Services.

## Conflict of Interest

VP, DG, Y-CL, DM, LC, JM, JA, and CC are employees of Becton, Dickinson and Company. The remaining authors declare that the research was conducted in the absence of any commercial or financial relationships that could be construed as a potential conflict of interest.

## Publisher’s Note

All claims expressed in this article are solely those of the authors and do not necessarily represent those of their affiliated organizations, or those of the publisher, the editors and the reviewers. Any product that may be evaluated in this article, or claim that may be made by its manufacturer, is not guaranteed or endorsed by the publisher.

## Footnotes

1
https://www.fda.gov/medical-devices/coronavirus-disease-2019-covid-19-emergency-use-authorizations-medical-devices/vitro-diagnostics-euas#individual-antigen
