# Supplementary figures and images for: Factors that Influence the Reported Sensitivity of Rapid Antigen Testing for SARS-CoV-2
Source: Front Microbiol. 2021 Oct 5;12:714242. doi: 10.3389/fmicb.2021.714242 (PMC8524138; doi:10.3389/fmicb.2021.714242)

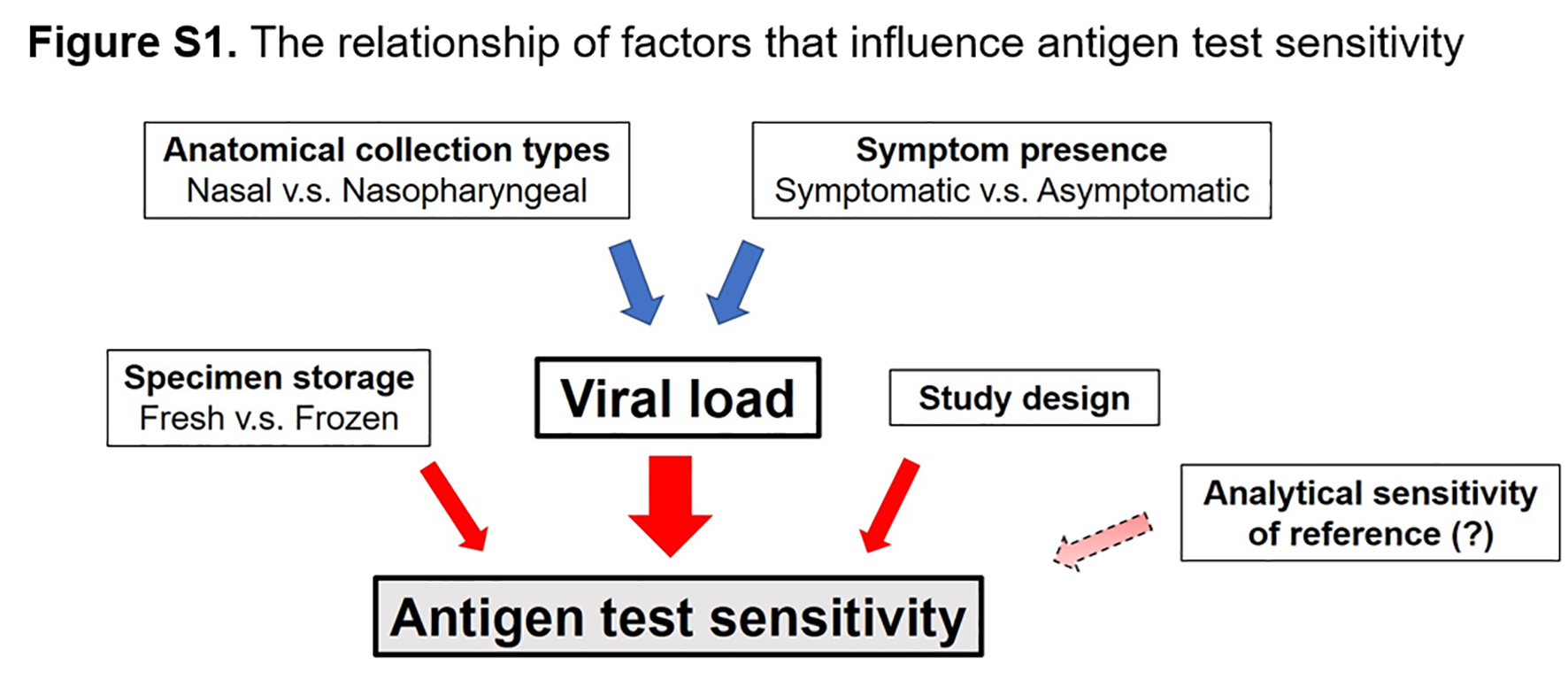

Supplement: Supplementary file 1 [file Image_1.JPEG]

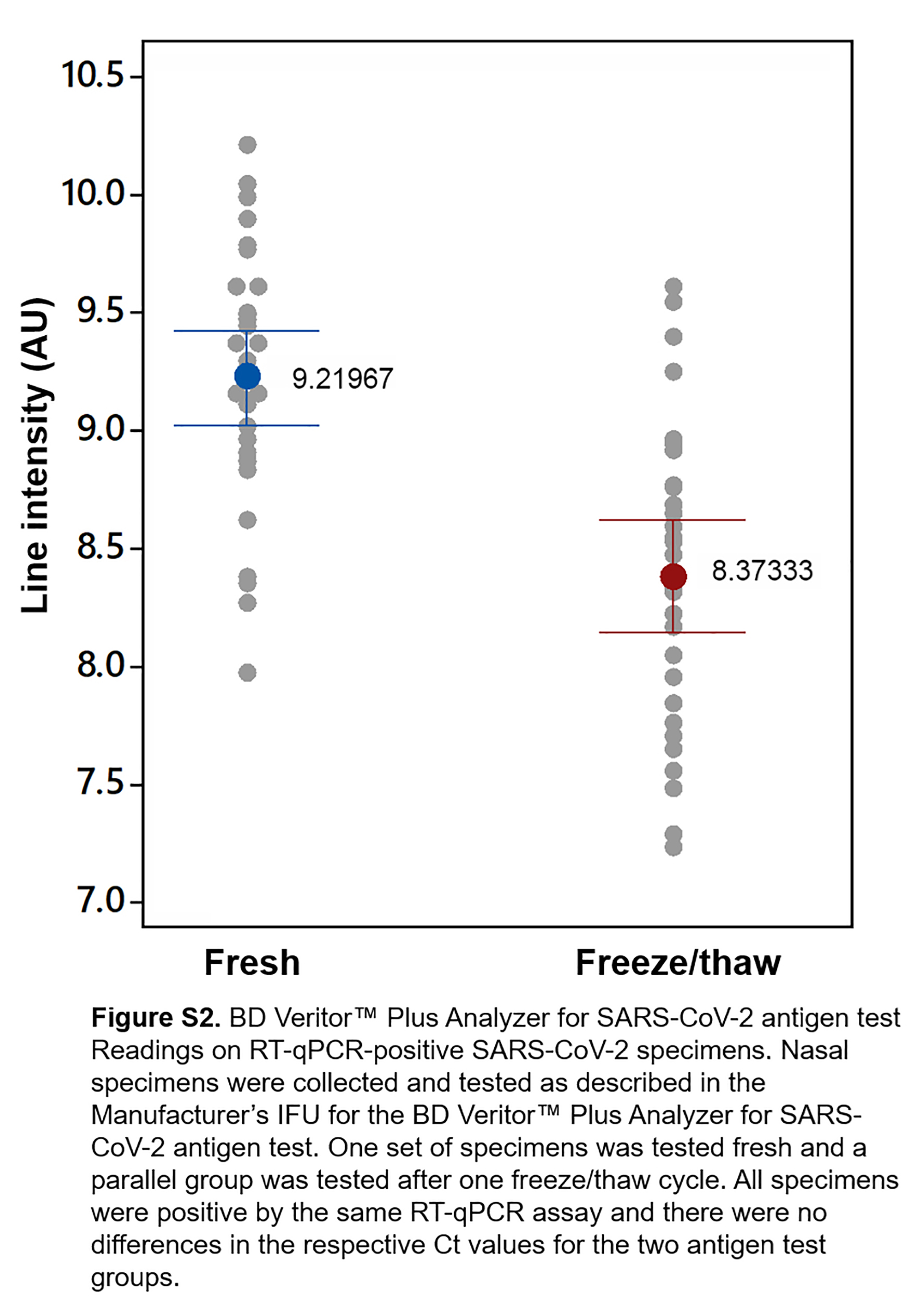

Supplement: Supplementary file 2 [file Image_2.JPEG]
