# Supplementary material for: Factors that Influence the Reported Sensitivity of Rapid Antigen Testing for SARS-CoV-2
Source: Front Microbiol. 2021 Oct 5;12:714242. doi: 10.3389/fmicb.2021.714242 (PMC8524138; doi:10.3389/fmicb.2021.714242)
Supplement: Supplementary file 3 [file Table_1.DOCX]

| **Table S1.** Antigen test sensitivity across brands (minimum number of studies = 3 to be included) | | | | |
| --- | --- | --- | --- | --- |
| **Antigen test name** | **No. data sets** | **Total Reference (+)** | **Sensitivity (%)** | **95% CI** |
| Abbott Panbio | 28 | 4,400 | 74 | [68, 80] |
| BinaxNOW | 14 | 1,113 | 75 | [64, 84] |
| Lumipulse | 4 | 295 | 70 | [48, 86] |
| LumiraDx | 6 | 331 | 97 | [85, 99] |
| Respi-Strip | 6 | 372 | 37 | [30, 44] |
| RIDA Quick | 4 | 172 | 57 | [40, 73] |
| Roche RAT | 6 | 614 | 65 | [56, 73] |
| Sofia | 5 | 296 | 79 | [62, 90] |
| Standard F | 5 | 1,091 | 56 | [48, 65] |
| Standard Q | 15 | 1,427 | 80 | [72, 86] |
| Veritor | 5 | 252 | 83 | [75, 89] |
| **Abbreviations:** CI, confidence interval | | | | |
|  | | | | |
