# Supplementary material for: Factors that Influence the Reported Sensitivity of Rapid Antigen Testing for SARS-CoV-2
Source: Front Microbiol. 2021 Oct 5;12:714242. doi: 10.3389/fmicb.2021.714242 (PMC8524138; doi:10.3389/fmicb.2021.714242)
Supplement: Supplementary file 4 [file Table_2.DOCX]

| **Table S2.** Experimental Factors affecting antigen test sensitivity | | | |
| --- | --- | --- | --- |
| **Independent variables** | **Increasing** | **Decreasing** |  |
| RT-qPCR result | High viral load | High Ct Score |  |
| Symptomology | Symptomatic | Asymptomatic |  |
| Reference specimen type | Nasal | NPS |  |
| Index storage condition | Fresh | Frozen |  |
| Spectrum bias | High bias | Low bias |  |
